# Acute Viral Meningitis of Unidentified Etiology: Insights from a Mixed-Methods Study

**DOI:** 10.3390/microorganisms13081747

**Published:** 2025-07-26

**Authors:** Andreea-Mădălina Panciu, Laura-Elena Marin, Ruxandra Moroti, Adriana Hristea

**Affiliations:** 1 Faculty of Medicine, Carol Davila University of Medicine and Pharmacy, 020021 Bucharest, Romania; andreea-madalina.firtat@drd.umfcd.ro (A.-M.P.); laura-elena.stoichitoiu@drd.umfcd.ro (L.-E.M.); ruxandra.moroti@umfcd.ro (R.M.); 2 “Prof. Dr. Matei Bals” National Institute for Infectious Diseases, 021105 Bucharest, Romania; 3 Colentina Clinical Hospital, 020125 Bucharest, Romania

**Keywords:** viral meningitis, etiologic diagnosis, mixed-methods study, diagnostic challenges

## Abstract

Background: Etiologic diagnosis in suspected viral meningitis is not always achievable, yet it can play a significant role in patient management. Our study aimed to do a comprehensive analysis of current practices regarding etiologic diagnosis in these cases and compare patients with and without known etiologic diagnosis in order to visualize if and how having an etiological diagnosis can impact patient management and/or outcome. Methods and results: We conducted a convergent mixed-methods study. Quantitative data was obtained from 118 patients hospitalized during a one-year period. There were 40.7% (*n* = 48) cases with unknown etiology. The length of hospitalization was longer in the group with unknown etiology vs. known etiology (12.6 days vs. 9.8 days *p* = 0.01). Thematic analysis was used for the qualitative approach in order to evaluate physicians’ overall perceptions regarding this subject. Conclusions: Our mixed-methods study shows that while clinicians consider etiologic diagnosis very important, it remains a diagnostic challenge even in modern times. Continued efforts are needed to optimize diagnostic strategies and address existing gaps in the etiologic workup of viral meningitis. There may be overlooked pathogens that have cost-efficient testing methods, like TBEV, that can be introduced in a testing protocol and may enhance patient management and reduce unnecessary hospital stays.

## 1. Introduction

The viral causes of meningitis and meningoencephalitis (ME) have been increasingly recognized due to the use of molecular diagnosis and the introduction of arboviral serologies [1,2,3]. Several arboviruses like West Nile virus (WNV) and tick-borne encephalitis virus (TBEV) have been associated with central nervous system (CNS) infections, and they are not included in the commonly used ME multiplex polymerase chain reaction (PCR) panels, but serological tests are available, adding value for diagnosis [1]. However, some pathogens are missed, and a proportion of infectious ME related to other viruses, parasites, fungi, and fastidious bacteria that require specific diagnostic investigations are categorized as aseptic meningitis of unknown etiology. Our primary objective was to investigate the underlying causes of meningitis or meningoencephalitis (ME) cases labeled as “viral” and to compare patients with a confirmed etiology to those with an unknown etiology, in order to determine whether an unidentified cause affected their management and/or clinical outcomes. A secondary objective was to explore the perspectives of infectious disease physicians who treated these patients, focusing on their views regarding the need for, and importance of, establishing an etiological diagnosis in suspected viral meningitis or ME, their preferred diagnostic methods, and their overall approach to patient management.

## 2. Materials and Methods

### Research Design

This study employed a modified convergent mixed-methods design [4]. Although the quantitative data were originally collected in 2023, they were retrospectively entered and analyzed concurrently with qualitative data collected in 2025. Both datasets were analyzed independently and given equal weight, with the aim of integrating the findings from both methods to provide a comprehensive understanding of the phenomenon. This design maintains the analytical parallelism characteristic of the convergent approach, despite the asynchronous data collection. We carried out a quantitative analysis of non-bacterial and non-fungal meningitis/ME cases admitted over a one-year period in a single medical unit. We conducted a qualitative study involving the infectious diseases doctors that treated these types of patients in order to gain insights into the physicians’ perceptions and opinions regarding the necessity and significance of identifying the cause of suspected viral meningitis/ME and the preferred diagnostic approaches.

In the end, the results generated from both approaches were compared in order to gain a more comprehensive understanding of the aspects we wanted to evaluate. We also aimed to evaluate the potential reasons and disagreements behind etiological diagnostic decisions and case management, as each clinician selects his own set of assays based on a complex cognitive process with multiple pros and cons behind their decisions. Personal experience is frequently used in this process of inference, which inherently links to a bias in prioritizing some tests over others in certain situations, resulting in a certain variance between the diagnostic tools employed, elements which are impossible to be evaluated through quantitative instruments alone.

## 3. Quantitative Approach

### 3.1. Study Design and Population

The quantitative study was a retrospective cohort study conducted in a monodisciplinary tertiary medical center, dedicated to infectious diseases, the Prof. Dr. Matei Balș National Institute for Infectious Diseases in Bucharest, Romania, with 680 beds for adults and children. Data for all pediatric and adult patients admitted from 1 January 2023 to 31 December 2023, who met the inclusion criteria according to the study protocol, were reviewed using electronic medical records.

Patients were eligible for inclusion if they had a clinical suspicion of infectious viral meningitis or meningoencephalitis (ME) and underwent lumbar puncture showing clear cerebrospinal fluid (CSF) with a leukocyte count greater than 5 × 10^6^ cells/L and lymphocytic predominance.

Clinical criteria of suspected meningitis were defined as the presence of acute onset of meningeal symptoms, including headache, fever, neck stiffness, photophobia, nausea or vomiting, and altered general condition. In addition, patients were classified according to the severity of their symptoms into subgroups: (1) mild—alert and oriented, with headache and low-grade fever but without neurological deficits or hemodynamic instability; (2) moderate—presenting with high fever, severe headache, nuchal rigidity, and systemic symptoms such as repeated vomiting or mild confusion; and (3) severe—showing altered mental status (stupor or coma), focal neurological signs (e.g., cranial nerve palsy, hemiparesis), seizures, or signs of hemodynamic instability.

Eligible cases required either confirmation of a viral etiology by PCR or serology, or negative bacterial and fungal cultures/PCR. In this study, “unknown viral etiology” was defined as meningitis/ME cases with CSF findings suggestive of a viral infection (clear CSF, lymphocytic pleocytosis) and exclusion of bacterial, fungal, and parasitic causes through appropriate microbiological testing, but in which no specific viral pathogen was confirmed by PCR or serology.

The exclusion criteria consisted of any identified bacterial, fungal, or parasitic cause including confirmed tuberculosis, rickettsiosis, Lyme disease, syphilis, brucellosis, cryptococcosis, toxoplasmosis for meningitis or ME, and HIV-positive patients.

### 3.2. Laboratory Diagnostic

The diagnosis was made using routine CSF tests, including microscopy, standard cultures, meningitis/ME multiplex-PCR (Meningitis/Encephalitis BioFire^®^ panel-BioFire Diagnostics, Salt Lake City, UT, USA) and serology.

The Meningitis/Encephalitis BioFire^®^ panel (BioFire Diagnostics, Salt Lake City, UT, USA) automatically extracts and processes the nucleic acids for enterovirus, herpes simplex virus type 1 and 2 (HSV1/HSV2), varicella-zoster virus (VZV), cytomegalovirus (CMV), human herpes virus 6 (HHV6) and human parechovirus, along with bacterial PCRs (*S. pneumoniae*, *H. influenzae*, *N. meningitidis*, *L. monocitogenes*, *S. agalactiae*) and fungal PCR (for *C. neoformans*).

Additionally, the following specific ELISA commercial antibody tests were used: CSF WNV IgM antibody detection by Focus Diagnostics (Cypress, CA, USA) and serum IgM antibody detection for WNV produced by Virion/Serion (Würzburg, Germany); serum IgM antibody detection for enteroviruses (ECHO-virus and Coxsackie virus) and mumps virus produced by Virion/Serion (Würzburg, Germany); serum IgM antibody detection for VZV and HSV1+2 produced by Bio-Rad (Marnes-la-Coquette, France); serum IgM and IgG antibody detection for TBEV produced by VIROTECH (Rüsselsheim, Germany).

### 3.3. Statistical Analysis

For the quantitative study, we used descriptive statistical methods for populations, calculating means, medians, standard deviations, minimum, maximum, and interquartile ranges for all numerical variables. We studied categorical variables using frequency analysis. In terms of inferential statistics, we used chi-squared multinomial tests to determine the association between categorical variables, *t*-tests, and their non-parametric counterparts to find associations between numerical and categorical variables. For all statistical tests, we considered a *p*-value of 0.05 for statistical significance. All tests were run in IBM SPSS Statistics, version 25.

## 4. Qualitative Approach

### 4.1. Methodology

For the qualitative analysis, we used semi-structured, in-depth interviews, with equal numbers of specialist physicians from the adult departments and pediatrics departments, from the institution where the included patients were hospitalized. The Consolidated Criteria for Reporting Qualitative Research were used to report the methodology of the qualitative design [5]. The sampling strategy was a convenient one, participant recruitment was directed face-to-face, and the information and the consent forms were sent via e-mail. The interview was based on eight questions which are shown in Table 1.

Additional questions were asked to ensure rich data collection. The questionnaire was initially piloted on one person to ensure the adequacy of the questions, with the subsequent interview not being included in the final analysis. No modification of the interview topic guide was made afterwards. The interviews were conducted face-to-face and they were audio recorded. All the interviews were transcribed verbatim by the interviewer, with the anonymization of the transcripts.

### 4.2. Sample and Data Collection

We enrolled 20 physicians. Afterwards, data saturation was achieved, and the inclusion process was stopped. The participants included were volunteers and did not receive any remuneration for their enrollment. Participants were not involved in the development of the research questions, study design, or the recruitment process. Data was collected at the workplace (“Prof. Dr. Matei Balș” National Institute for Infectious Diseases). During the interview, there was no one else present except the participant and the interviewer. The median duration of the interviews was around 15 min. After the publication of the article, all the audio recordings will be destroyed.

### 4.3. Analysis

We performed an experiential form of thematic analysis using an inductive, data-driven approach. We went through all the phases of qualitative research, beginning with data familiarization, followed by the development of initial codes, the identification and refinement of emerging themes, their subsequent definition and labelling, and finally, the synthesis of our findings into a comprehensive report [6]. Our themes incorporated not only codes that recur systematically, but also codes that align with the saliency analysis principle [7]. After familiarization with the data, the interviewer generated the codes and presented them to the last author, who was also the supervisor of the study. Then, the codes were matched into themes in around three meetings with all authors. The report was then written and sent to three randomly selected participants to perform member checking. We achieved data saturation after 20 interviews.

## 5. Ethical Approval

The study received approval from our Institutional Review Board committee (No. C07186/20 June 2023; C06727/28 June 2024).

## 6. Results

### 6.1. Quantitative Approach

Between January 2023 and December 2023, a total of 118 patients met the predefined inclusion and exclusion criteria. Of these, 59.3% (*n* = 70) were male, 57.6% (*n* = 68) belonged to the pediatric group (≤18 years), and 42.3% (*n* = 50) were adults. The median age in the pediatric group was 7 years (IQR 5.5–11), while in the adult group it was 51 years (IQR 33–62). Demographic and clinical characteristics of patients with confirmed and unconfirmed viral etiology are presented in Table 2.

A viral etiology was identified in 59.3% (*n* = 70) of patients overall, with a significantly higher detection rate in children (67.6%, *n* = 46) compared to adults (48%, *n* = 24; *p* = 0.04). The distribution of detected pathogens was as follows: enteroviruses in 71.4% (*n* = 50), West Nile virus (WNV) in 17.1% (*n* = 12), varicella-zoster virus (VZV) in 7.1% (*n* = 5), herpes simplex virus type 1 (HSV-1) in 2.8% (*n* = 2), and mumps virus in 1.4% (*n* = 1). Of all included patients, 83.9% (*n* = 99) underwent meningitis/ME multiplex PCR testing, yielding a positive result in 50.5% (*n* = 50). Among these positive samples, 80% (*n* = 40) were enteroviruses, 2% (*n* = 1) showed co-detection of enterovirus and HHV-6, 6% (*n* = 3) were positive for HHV-6 alone, 8% (*n* = 4) were positive for VZV, and 4% (*n* = 2) for HSV-1.

Patients with negative meningitis/ME multiplex PCR (*n* = 44) and those in whom meningitis/ME multiplex PCR was not performed (*n* = 19) were tested with different serologies, with positive results in 32.8.% (*n* = 23) of these patients. Among 23 patients with retrieved etiology due to serologic tests, 52.1% (*n* = 12) were diagnosed with WNV infection, 39.1% (*n* = 9) with enteroviral infection, and 4.3% (*n* = 1) with mumps meningitis and VZV. Of 8% (*n* = 10) patients tested for TBEV, none had a positive result.

Enterovirus was the most frequent etiology, accounting for 71.4% (*n* = 50) among 70 patients with detected etiology. Enterovirus was mainly detected using meningitis/ME multiplex PCR in 41 patients, including codetection with HHV6 in one sample. The detection of HHV6 by meningitis/ME multiplex PCR was concomitant with another cause of viral meningitis, enterovirus (*n* = 3) or VZV (*n* = 1).

In the group with known vs. unknown viral etiology, the median age was 11 vs. 26.5 years (*p* = 0.15), and the male/female ratio was 1.5 vs. 1.4, respectively.

A substantially higher proportion of adults had an unidentified etiology compared to children, with 53.0% of adults (*n* = 27) lacking a determined cause versus only 31.4% of children (*n* = 21) (*p* = 0.02). Among patients with an unknown etiology, 23 (47.9%) had meningitis and 25 (52.1%) had ME. In contrast, among patients with a confirmed etiology, 51 (72.9%) were diagnosed with meningitis and 19 (27.1%) with ME (*p* = 0.01).

The median length of hospitalization was 7 (IQR 6–12) days, and it was longer in the group with unknown etiology vs. the group with known etiology (12.6 days vs. 9.8 days *p* = 0.01) and in adults vs. children (10 days vs. 7 days, *p* < 0.001).

A total of 11.8% of patients (*n* = 14) required ICU admission. Among these patients, 10.4% (*n* = 5) were from the group without an identified etiology vs. 12.8% (*n* = 9) from the group with known etiology (seven of them with WNV).

Regarding clinical outcomes, at discharge, 70.3% (*n* = 83) of patients achieved full recovery, while 18.6% (*n* = 22) showed partial recovery, 11.4% (*n* = 8) required transfer to other medical facilities for continued care, and 4.2% (*n* = 5) died. All fatalities occurred in the adult group: four were associated with West Nile virus infection (three men aged 61, 75, and 82 years, and one woman aged 87 years) and one with varicella-zoster virus infection (a woman aged 67 years).

### 6.2. Qualitative Approach

For the qualitative part of the study, we interviewed 20 physicians. Their age ranged from 29 to 59 years old. All of them were infectious diseases specialists, 12 were senior physicians, and w had also a second specialty in pediatrics. There were 12 physicians from the pediatric departments and 8 physicians from the adult departments. Of the responders, 85% were female physicians, in accordance with the male-to-female physician ratio in our institution.

After analyzing and coding the transcripts, we identified three themes, which are defined in Table 3: “Perceived importance of etiology in suspected viral meningitis/ME—A range of justifications”, “The challenges of establishing an etiological diagnosis in suspected viral meningitis/ME”, and “Knowledge on TBEV in Romania: gaps in testing, clinical suspicion and diagnosis”.

#### 6.2.1. Perceived Importance of Etiology in Suspected Viral Meningitis/ME—A Range of Justifications

The perceived importance of establishing an etiology in suspected viral meningitis/ME is associated with various rationales. The main reasons for which physicians consider the etiological diagnosis important are related to its impact over treatment decisions, the extent of investigations, prognosis, and length of hospitalization. In addition, the value that participants attribute to the etiology determination is closely related to the fact that it permits them to rule in or rule out the few infections that have specific treatments (mainly, HSV-1, HSV-2, and VZV), as it illustrated below.

*“There are a few viruses for which there is etiological treatment, and it would be worth identifying or at least ruling them out. I’m referring to herpes virus, CMV, VZV—antivirals can also be used in HHV6 meningoencephalitis—so at least these should be ruled out.”*

(Physician 7)

*“In a meningitis caused by Coxsackie, a meningitis with paramyxovirus, a meningitis with Armstrong virus, I’m not that interested in the etiology because the treatment is the same. If I’m talking about influenza meningitis or herpetic meningitis, then yes, I do need the etiology because there I can support an etiological treatment, justify it, and also obtain benefits from it.”*

(Physician 12)

This further leads to a reduction in the duration of empirical treatment, as well as the risk of adverse effects from unnecessary medications. Moreover, it limits extensive and sometimes emotionally distressing investigations that tend to be done in the absence of a clear etiological diagnosis. Furthermore, having a clear etiology for meningitis and ME is recomforting and therefore it makes physicians more confident when it comes to shortening the number of days that patients spend in the hospital. In turn, this decreases overall hospitalization costs and the risk of healthcare-associated infections, which offers them an augmented sense of value, as is further shown in the following.

*“It’s a diagnosis that should be made fairly quickly because otherwise you’re forced to stay under broad-spectrum coverage, which is harmful because of… uh, high costs, because it traumatizes the patient—you poke today, and again tomorrow, to see what’s going on, the day after tomorrow too… uh, there are high costs both for investigations and for additional medication, plus the impact this umbrella-type therapy has, the side effects that may occur—they do exist, it’s not really that simple to just give everything and see what happens (…)”*

(Physician 20)

*“A clearly established etiological agent can decide how long we give treatment, how long we keep the patient hospitalized, so that we discharge them safely, without the symptoms reappearing.”*

(Physician 5)

*“Sometimes we hospitalize the patient for weeks, we don’t know the cause, and that means a risk of healthcare-associated infections.”*

(Physician 12)

The etiological diagnosis is considered more important and urgent if the case is more severe and/or the patient’s condition is deteriorating: *“For sure, if I were dealing with a severe case, I would keep looking until I found clearly what it was, I wouldn’t stop…”* (Physician 13)

In contrast, in cases with a more favorable and rapid favorable evolution, physicians tend to limit further investigations, considering the disease as self-limiting, which poses no supplementary risks in their view, as this doctor clearly emphasizes: *“Of course, it depends a lot on the patient, if things are very clear and it seems clearly like a [specific] viral etiology, let’s say there was diarrhea, there were digestive symptoms, it suggests, let’s say, an enterovirus, yes, and the patient evolved well and so on, I don’t think it makes sense to turn ourselves upside down with sophisticated stuff.”* (Physician 12)

Most of the interviewed doctors mentioned the importance of the etiological diagnosis from the psychological and emotional perspective of the in-hospital usual trio: the physician, the patient, and the patient’s family. They consider that establishing an etiology with certainty is associated with an increase not only in patients’, but also their family members’, level of trust in the healthcare system, the medical act, and their physicians, which ultimately leads to an improvement in the doctor–patient relationship. Thus, the absence of an etiological diagnosis can generate anxiety on all sides. On one hand, physicians tend to continue investigations more assiduously in the search of the precise cause of the disease to confirm their suspicions, and on the other hand, when confronted with the uncertainty of the disease, the patient/patient’s family are asking for the closure that is found in having a precise diagnosis from an etiologic point of view, as illustrated below.

*“The moment you have an etiology, you breathe a bit easier, like okay, I have an etiology, and you make decisions more quickly, so to speak—you no longer wait, or keep thinking about it.”*

(Physician 11)

*“I would need an answer in a severe case, because people, especially if something like deaths happens, they need to be at peace with it, they need to know what happened, it’s very important to have closure.”*

(Physician 13)

*“But it influences the relationship with the patient, because uh, they have more trust in us as professionals once we’ve discovered exactly the etiological agent.”*

(Physician 18)

*“In general, that’s what parents usually ask you. There’s a focus on this part. WHICH VIRUS WAS IT?”*

(Physician 3)

Besides all the above, etiological diagnosis was also considered important from an epidemiological point of view, as to understand viral circulation and recommend appropriate prophylaxis.

*“I would have some concrete epidemiological data, and I know what the situation is and I can monitor it easily, and third, because in this way, based on real data, I can promote vaccination.”*

(Physician 12)

#### 6.2.2. The Challenges of Establishing an Etiological Diagnosis in Suspected Viral Meningitis/ME

Aseptic meningitis and ME are seen by physicians as being more challenging than bacterial meningitis when it comes to establishing a certain etiological diagnosis, mostly due to the wide spectrum of potential causes and the inherent higher costs of specific investigations, which, in turn, frequently makes them unavailable.

*“Viral meningitis can have multiple etiologies, and compared to bacterial etiology testing where we can usually get a culture in a few days, in these cases is much harder to establish the specific cause, because we need to run various tests that unfortunately come with significant higher costs compared to suspected bacterial meningitis where cultures are much cheaper…”*

(Physician 17)

However, physicians suggest that clinical presentation can be useful in guiding the selection of appropriate tests for each individual case before turning to various modern and extensive laboratory diagnostic techniques. Combining several diagnostic methods increases the chances of establishing an etiological diagnosis, but their use should be tailored according to the specific case to improve overall patient management, as illustrated below.

*“(…) there are situations where we can orient ourselves towards the etiological diagnosis based on the patient’s clinical picture; for example, if the patient has varicella, then we know, we assume that most likely the varicella virus is the one that caused the meningeal or encephalitic condition. Or if, I don’t know, there are digestive symptoms, then we think of an enterovirus.”*

(Physician 9)

Most physicians consider a meningitis/ME multiplex PCR panel as the preferred method for etiological diagnosis, due to its rapid results and the wide range of pathogens detected. However, they also mentioned a few disadvantages: significant costs, limited availability in some clinical settings and co-detection of viruses that are probably not responsible for the clinical manifestations (e.g., HHV-6, CMV), the latter one leading to an over-investigation of the patient in order to rule out unanticipated differential diagnostics. In addition, they acknowledge the fact that the M/ME multiplex PCR panel has reduced sensitivity compared to singleplex PCR in cases with low viral load (qualitative vs. quantitative), and the lowest sensitivity for one of the most feared pathogens—HSV. Physicians also mentioned the fact that from a therapeutic perspective, identifying HSV could be more effectively achieved by using only a quantitative HSV-1/2 singleplex PCR.

*“The PCR panel for viral infections of the nervous system has made our lives much easier in recent years. I don’t even think we remember what it was like before PCR—back then we’d just diagnose clear fluid meningitis, probably viral […] Now we can perform many tests that allow for a much more accurate etiological classification.”*

(Physician 6)

*“The BioFire panel we use has this issue where its lowest sensitivity is actually for cryptococcus and herpes simplex.”*

(Physician 12)

Serological tests are also considered extremely useful, especially as they complete the most frequent list of pathogens by testing for arboviruses like West Nile virus (endemic in Romania) and TBEV. Serologies are seen as more accessible, low-cost, and less invasive when performed from serum. However, their limitations mentioned include long time for results, unavailability of the tests in some clinical settings, cross-reactivity, the need for paired samples, and dependence on the timing of sample collection and on the individual immune response. The choice for the use of arboviral serologies should be guided by the patient’s epidemiological context.

*“When it comes to serologies, you always must think carefully, because the patient may have been exposed to multiple viruses throughout their life, we don’t know the exact antibody titer, and the patient’s responsiveness may be different. Secondly, there’s the specificity of serological tests, as there are often cross-reactions, and thirdly, there’s the specificity of the diagnosis at that moment, because the patient may have an antibody titer, but if there isn’t a dynamic increase in antibodies to indicate an acute infection, it may end up being a retrospective diagnosis, which doesn’t help us at all. Plus, in viral meningitis, since it can present with a severe clinical picture, you can’t really afford to wait for paired serum samples to detect antibodies.”*

(Physician 17)

*“I would systematically look for TBEV [serology] in any patient with viral encephalitis that is not herpetic and who is young…because, if in the case of West Nile, I would orient myself toward a patient with risk factors for neurological involvement, here, where I have an encephalitic component and no clear cause, and nothing shows up on BioFire and so on, that’s where I would expect it, especially when I have a lot of inflammation in the CSF—BioFire doesn’t find it, it doesn’t…”*

(Physician 12)

Additional methods for expanding the diagnostic panel were also mentioned in cases where commonly used tests (meningitis/ME multiplex PCR, serology) yield no results—for example, rapid antigen tests, singleplex PCR, or multiplex PCR panels from other sample sites (nasopharyngeal, digestive).

#### 6.2.3. Knowledge on TBEV in Romania: Gaps in Testing, Clinical Suspicion, and Diagnosis

In physicians’ opinions, TBEV is rarely tested in Romania due to several factors: low awareness among healthcare professionals (including infectious disease specialists), limited epidemiological studies, and very few reported cases. The absence of epidemiological data negatively impacts physicians’ perceptions of the TBEV infection prevalence in Romania. This leads to the exclusion of this possible etiology in patient evaluations. Doctors report that TBEV testing is not performed in their hospitals outside study protocols, and many are unaware that tests even exist in their hospital setting, as it is pointed out in the following.

*“On the other hand, the fact that it seems to have low epidemiological prevalence in Romania has influenced the decision to test for it.”*

(Physician 2)

*“Sure, it’s not very common, but we’re not even looking for it to know how common it is. That’s why it’s important to be aware, to know that there are epidemiological cases, because if I come and ask 〈How many cases of TBEV are there per 100,000 meningitis cases?〉, well, then I’ll assume the risk of it is negligible, so why bother with vaccination… but if I present supporting data and actual case reports, often with central nervous system sequelae, then I think it becomes very useful to know both the diagnosis and to have a case surveillance method.”*

(Physician 12)

Another mentioned barrier was the widespread association—both in the general population and among physicians—between tick bites and Lyme disease alone, leading to the neglect of other tick-borne illnesses, such as TBEV:

*“Regarding ticks, we’ve all focused only on Lyme disease.”*

(Physician 7)

From a therapeutic perspective, establishing an etiological diagnosis of TBEV can influence clinical management despite the absence of specific treatment, by preventing unnecessary antimicrobial use, supporting prognosis estimation, and offering psychological closure for both physicians and patients. TBEV testing is often considered by physicians, when no clear etiology is found through standard tests, the case is severe or progressive or/and there is clear epidemiologic risk. Perceptions varied from testing for TBEV as a last resort when no etiology is found, to testing it up front in encephalitic patients once herpetic causes have been excluded.

*“It changes how we think. It helps us anticipate disease progression, explain outcomes to families, and even advocate for vaccination.”*

(Physician 6)

The introduction of TBEV vaccination in Romania has slightly improved awareness among physicians and the general population. However, improving awareness of TBEV among healthcare providers and the public is considered essential.

*“Since vaccination was introduced, it seems to me that it’s a topic brought up more often, especially because we have a prophylactic method, and I think this has encouraged the discussion (…).”*

(Physician 2)

*“I think there is very little… knowledge. I honestly admit that I… rarely think of this etiology when I see a patient. I haven’t really encountered it, and because it’s a rare thing, I think of it just as rarely, unfortunately.”*

(Physician 3)

## 7. Discussion

In our quantitative observational one-year retrospective study, we describe the etiology of viral meningitis/ME among patients with assumed infectious, non-bacterial/non-fungal meningitis/ME who were hospitalized in a tertiary infectious disease hospital.

When more diagnostic methods were combined—meningitis/ME multiplex PCR in CSF and serologies, viral etiologies were detected in more than half of all cases, similar to other studies; however, the cause remained unidentified in a large proportion of patients 40.7% (*n* = 48) [3,8,9].

In a study performed in the U.K., of the total number of 683 patients with meningitis, 42% had unknown etiology [3]. When comparing our findings with those reported by McGill et al. in their multicenter U.K. cohort of adults with viral meningitis, several similarities and differences emerge. In both studies, enteroviruses represented the leading cause of confirmed viral meningitis, accounting for 71.4% (*n* = 50) of cases with a known etiology in our cohort and 80% in the U.K. series. However, a striking difference lies in the contribution of West Nile virus (WNV), which represented 17.1% of identified etiologies in our population but was not reported in the U.K. study, reflecting regional variations in arboviral circulation. Our cohort also included a substantial pediatric population, in whom the detection rate of viral etiology was significantly higher than in adults (67.6% vs. 48%, *p* = 0.04), whereas McGill et al. focused solely on adults. A higher proportion of adults in our series had an unidentified etiology compared with children (53.0% vs. 31.4%, *p* = 0.02), a finding consistent with the U.K. data, where approximately half of the adult cases lacked a confirmed cause. Notably, we observed longer hospitalizations overall, particularly among adults and patients with unknown etiology, and a higher mortality rate (4.2%), largely driven by severe WNV infections in older adults. These differences underscore the impact of local epidemiology, patient age distribution, and diagnostic availability on the observed etiology, clinical course, and outcomes of viral meningitis.

When comparing our cohort with the nationwide Spanish study by De Ory et al. (2013), several important similarities and differences emerge [8]. De Ory et al. prospectively studied 582 patients with viral infections of the central nervous system. Similarly to our findings, enteroviruses were the most common etiology, identified in 46.2% of their cases, compared with 71.4% (*n* = 50) in our cohort. Herpes viruses were also frequent in both studies: De Ory et al. reported 14.8% VZV and 7.4% HSV, whereas in our series, VZV accounted for 7.1% (*n* = 5) and HSV-1 for 2.8% (*n *= 2) of identified etiologies. A notable difference lies in the detection of WNV, which represented 17.1% of etiologies in our population but was not reported in the Spanish cohort, likely reflecting geographic differences in arboviral circulation. Regarding age distribution, De Ory et al. found that 61% of their patients were children (<15 years), closely paralleling our cohort in which 57.6% (*n* = 68) were pediatric cases. In both studies, molecular methods (particularly PCR) were central to the diagnostic approach, complemented by serology, although our series incorporated a multiplex PCR platform more extensively. Overall, both cohorts highlight enteroviruses as the dominant cause of viral CNS infections, while differences in the prevalence of specific pathogens, such as WNV, underscore the impact of regional epidemiology, diagnostic strategies, and population structure on observed etiologic patterns.

A considerable proportion of our cohort (40.7%) had no identified viral etiology despite comprehensive testing, and several factors may account for this. First, the diagnostic panels used may not have covered all potential neurotropic viruses. For example, parechoviruses, adenoviruses, arboviruses other than West Nile virus (such as Usutu virus or Toscana virus, both reported in parts of Europe), and even less common enterovirus serotypes were not systematically included in routine testing and could have been missed. Second, the timing of cerebrospinal fluid collection can critically influence diagnostic yield; low viral loads early in infection or rapid clearance before sampling may lead to false-negative PCR results. Similarly, for viruses requiring serologic confirmation (e.g., Epstein–Barr virus, or mumps virus), the absence of paired acute and convalescent sera may have limited detection. It is also possible that noninfectious conditions mimicking viral meningitis or atypical presentations of known pathogens contributed to the group without a defined etiology. Future studies integrating expanded multiplex panels, next-generation sequencing approaches, and serial CSF or blood sampling will be essential to reduce the proportion of unknown cases and to further elucidate the full spectrum of viral causes of meningitis and meningoencephalitis in our region.

Our quantitative study results showed that patients with a known etiology had a statistically significant lower duration of hospitalization compared to patients with unknown etiology (12.6 days vs. 9.8 days *p* = 0.01). Similarly, the study performed in the U.K. showed that establishing a clear etiology in terms of specific viral agents reduces inappropriate antibiotic and antiviral usage, length of hospital stays, and hospital admission costs [3]. We can correlate this information with the results from our qualitative approach, where the importance of establishing a clear etiology when it comes to treatment decisions overall, the extent of investigations, and the duration of hospitalization is clearly stated.

Regarding sensibility and specificity of currently used diagnostic tests, the meningitis/ME multiplex PCR panel BioFire (14 pathogens in approximately one hour) has rapid, comprehensive results and an overall 94.2% sensitivity and 99.8% specificity [10]. A meta-analysis including 19 studies obtained the following results regarding sensibility and specificity of meningitis/ME multiplex PCR panel BioFire: for all bacteria, sensitivity was estimated at 89.5%, and specificity at 97.4%, and for HSV-2, EV and VZV, sensitivities were between 75.5 and 93.8%, and specificities above 99%, and sensitivities for *L. monocytogenes*, *H. influenzae*, *E. coli*, and HSV-1, were suboptimal [11].

Our qualitative study results show the advantages and disadvantages perceived by the physicians in choosing the different diagnostic tools available, the preference for meningitis/ME multiplex PCR panel when available, but also the need for serologies in covering the common arboviruses not included in the spectrum of the PCR. Physicians emphasized the need for a stepwise approach to investigations, as well as integrative correlation of available tests to optimize medical decision-making in terms of both cost-efficiency and reducing the psychological impact on the patient.

As we can see from our quantitative study, the second cause of viral meningitis/ME found in our patients was WNV infection, which represented 17.1% (*n* = 12) of all cases with detected etiology in our study, but half of cases confirmed using antibody detection tests, which is the only available method of diagnosis, since WNV is not included in the meningitis/ME multiplex PCR panel. For almost three decades, WNV infections have been documented in the human population in Romania, with two major outbreaks occurring in 1996 and 2017–2018 [12,13]. The cross reactivity of flaviviruses, including WNV and TBEV, is well known, and their confirmation requires a neutralization assay or testing CSF for specific IgM and IgG [14].

Studies taking place in the neighboring countries of Romania (e.g., Serbia), which searched for causes among unknown etiology meningitis/ME cases, have unexpectedly identified TBEV cases (*n* = 3/15) [15]. In a recently published hospital-based study from Strasbourg, Alsace region, France, from 462 pediatric patients with neurologic syndromes only 26.4% were tested for IgM and IgG serum antibodies for TBEV, but retrospective testing of IgM and IgG CSF revealed four additional cases that were missed by not performing the serologic screening for TBEV [16] more often.

The role of TBEV in meningitis/ME cases in Romania is unknown, probably being underdiagnosed and underreported [17]. We can see from our qualitative study results that there are gaps in testing, clinical suspicion, and diagnosis when it comes to TBEV. In our quantitative study, the attending physicians requested TBEV serology in only 8% (N = 10) of patients from the total cohort; none of them had a positive result. As we correlate these results with our quantitative study, we can see that the reasons behind this decision are based on low awareness among healthcare professionals, limited epidemiological studies, and very few reported cases.

This study has several limitations that should be acknowledged. Being conducted in a single setting with a relatively small sample size, the findings may not fully capture the diversity of clinical practices or the perspectives of all physicians managing meningitis and meningoencephalitis. In addition, as a qualitative component was included, the views expressed may reflect the experiences of the participants rather than the wider medical community. Diagnostic testing was based on the tools available at the time of the study, which may have limited the ability to detect certain pathogens and contributed to the proportion of cases with unknown etiology. Future research should aim to address these limitations by conducting multicenter studies that encompass broader geographic and demographic populations, increasing sample sizes to improve representativeness and statistical power, and incorporating more advanced diagnostic technologies such as next-generation sequencing or expanded multiplex panels. Such efforts would enhance understanding of the etiology and management of viral meningitis and meningoencephalitis and could guide the development of more targeted diagnostic and therapeutic strategies.

## 8. Conclusions

Our study showed that there is still a large proportion of patients with no identified cause when it comes to suspected viral meningitis/ME and that etiological diagnosis may improve the duration of hospital stay and possibly overall patient management. Physicians’ perceptions appear to seek a balance between the need for an etiologic diagnosis in a potentially serious condition like ME requiring tailored management and the goal of providing cost-effective, patient-centered care. Developing a protocol for establishing the etiological diagnostic in cases of suspected viral meningitis/ME could be extremely useful, as diagnostic decisions are shaped by case severity, availability of modern tools such as meningitis/ME multiplex PCR, and the clinicians’ familiarity with various pathogens.

In the case of TBEV, limited public awareness, insufficient epidemiological data, and systemic challenges such as the unavailability of diagnostic tests can contribute to the under-recognition of potentially severe infections. Strengthening diagnostic infrastructure, promoting continuous medical education, and integrating epidemiologic context into clinical reasoning are essential steps toward improving patient care and guiding preventive strategies such as vaccination in Romania.

## Figures and Tables

**Table 1:** Interview topic guide.

1. How often do you treat suspected viral meningitis/ME and what difficulties or dilemmas do you face in managing these patients?
2. What is your opinion regarding the investigation of the etiological diagnosis in these cases of serous meningitis suspected to be viral?
3. What diagnostic tool do you usually use in these cases and why?
4. Have you ever encountered cases of TBEV meningitis/ME?
5. When do you consider testing for TBEV in suspected cases of viral meningitis/ME?
6. Have you encountered difficulties in testing for TBEV in these cases?
7. Do you think that the etiologic diagnosis of TBEV is important in managing a case of suspected viral meningitis/ME?
8. What do you think about the awareness regarding TBEV in Romania?

**Table 2:** Demographic and clinical characteristics of patients with confirmed vs. unconfirmed viral etiology.

Variable	Confirmed Viral Meningitis/ME *n*= 70	Meningitis/ME with Undetected Etiology *n* = 48	*p*
Male: Female	1.5	1.4	0.8
Age groups (years) *n* (%)			0.5
0–18	46 (65.7)	21 (43.7)	0.02
19–99	24 (34.3)	27 (56.3)	
Median age (IQR)	11 (6–33)	26.5 (8–51)	0.15
No of cases of meningitis *n* (%)	51 (72.9)	23 (47.9)	0.01
No of cases of ME *n* (%)	19 (27.1)	25 (52.1)	
Length of hospitalization (days) Mean (standard deviation)	9.8 (9.7)	12.6 (10.2)	0.01
ICU admission *n* (%)	9 (12.9)	5 (10.4)	0.8
Death *n* (%)	5 (7.1)	0	0.2
Season *n* (%)			0.4
Summer (June–August)	39 (55.7)	20 (41.7)	0.18
Spring (March–May)	8 (11.4)	5 (10.4)	
Fall (September–November)	20 (28.6)	13 (27.1)	
Winter (December–February)	3 (4.3)	10 (20.8)	

ICU—intensive care unit; /ME—meningoencephalitis.

**Table 3:** Overview of themes.

Themes Title	Themes Definitions
6.2.1. Perceived importance of etiology in suspected viral meningitis/ME—A range of justifications	This theme highlights the importance that physicians attribute to establishing an etiological diagnosis in suspected viral meningitis/ME, as it guides the treatment, limits many unnecessary investigations, and offers a prognosis. Furthermore, it plays a key role in providing clarity for physicians/patients and supporting trust in medical decisions.
6.2.2. The challenges of establishing an etiological diagnosis in suspected viral meningitis/ME	This theme explores the challenges that physicians face when dealing with cases characterized by complex etiologic diagnostic challenges, due not only to the broad spectrum of potential etiologies, but also to the high costs that characterize the specific investigations and their lack of availability.
6.2.3. Knowledge on TBEV in Romania: gaps in testing, clinical suspicion and diagnosis	This theme reflects the limited awareness and testing of TBEV in Romania, largely due to a lack of epidemiological data, poor clinical suspicion, and lack of available tests.

## Data Availability

The original contributions presented in this study are included in the article. Further inquiries can be directed to the corresponding author.
